# Simulation approach to interprofessional education

**DOI:** 10.1093/geroni/igab046.402

**Published:** 2021-12-17

**Authors:** Margaret Greenwald, Jennifer Mendez

**Affiliations:** 1 Wayne State University, Wayne State University -, Michigan, United States; 2 Wayne State University, Detroit, Michigan, United States

## Abstract

A team practice simulation approach to interprofessional education is presented. Participants (79 trainees over 4 years) were assigned to one of six teams representing clinical services for a client with complex clinical needs (medical care, outpatient therapy, dental, nutrition, speech and hearing, leadership). Each student within the team was assigned a specific role (e.g., primary care, policy maker, family member). A critical component of this activity is that each participant adopted the role and perspective of an individual in a different clinical area than their own. In preparation for a live discussion by all participants, each team met to study their assigned clinical roles and to prepare a one-page slide addressing specific questions given only to their team. At the live session, the overall goal was to develop a coherent clinical plan for the client. This is an effective approach for IPE in care of clients across the lifespan.

